# Supplementary material for: Chromothripsis during telomere crisis is independent of NHEJ, and consistent with a replicative origin
Source: Genome Res. 2019 May;29(5):737–49. doi: 10.1101/gr.240705.118 (PMC6499312; doi:10.1101/gr.240705.118)
Supplement: Supplemental Material [file supp_gr.240705.118_Supplemental_file_1.zip › contigs/annotated_contigs/DB37/contig.3.DB37_length_663_mean_cov_7.89592760181.docx]

**DB37_length_663_mean_cov_7.89592760181**

```
TTATACTCTTTTAGTTATTTTTTAATGTACACAAATTATT|TTACCA|CAGTCACCCTGTTGTGCTAGCAAATACTAGGTCTTATTCAT
 >chr4:115271760-115271800 + E=3e-03 p=4e-03   >chr14:32647701-32647951 + E=1e-138 p=5e
TCTTTCTAATTATTTTTTGTACGCATTAACCCCACTTCTTATTCACCCTTCCCACCATTACCCTTCTCAGTCTTTGGTAACCATCCTTC
-02
TATACTCTATCTCCATGAGTTCAATTGTTTTAAATGTTAGCTCCCCCAAAGAGTGAAAACATGTAGTTTGTCTTTCTGTGCAAGTATTA

TTTCACTTAACATGATGACCTCTGGTTCC|AT|TGCAAATACTAAATGCAATCTTGTATGTAAAATACCTGTCAAGTATTTGTTGATTA
                              >chr14:32666359-32666728 + E=3e-210
ATAAATATTACACTCTTCCACCACATTTTCTAACTCTTGAGTTATTACCTCTAGGAAAGAATAAATATAATGATACACAAATTCCAGGA

ACTGAAGTTTTCCGTATCAGCCTATACAGTGTTCCTCAGACTAGGATCTAGAACCTCTTGGAGTCTTCAGACAGATTCTTAAGGGTTAG

AGGATTCTCTAGAGGATTTTTAGAAATAGTTTTTTATTGGGATGATCATCTTAAAATATTAAAATAGGCAGGGTCTAAAACATCTGAAT

ATTCACCTTATAACTAAATATTGTCATGAGATTTCAACATGAAT
```
